# Efficacy of botulinum toxin in modifying spasticity to improve walking and quality of life in post-stroke lower limb spasticity - a randomized double-blind placebo controlled study

**DOI:** 10.1186/s12883-019-1325-3

**Published:** 2019-05-11

**Authors:** Anupam Datta Gupta, Renuka Visvanathan, Ian Cameron, Simon A. Koblar, Stuart Howell, David Wilson

**Affiliations:** 1 Central Adelaide Rehabilitation Services, The Queen Elizabeth Hospital, 28 Woodville Road, South Australia- 5011, Adelaide, SA 5005 Australia; 2 National Health and Medical Research Council Centre of Research Excellence in Frailty and Healthy Aging. The Queen Elizabeth Hospital, University of Adelaide, 28 Woodville Road, South Australia- 5011, Adelaide, SA 5005 Australia; 3 Head John Walsh Centre for Rehabilitation Research, Sydney Medical School, University of Sydney, Sydney, NSW 2006 Australia; 4 South Australian Health & Medical Research Institute (SAHMRI), GPO Box 11060, Adelaide, Adelaide, SA 5001 Australia; 5 University of Adelaide, Adelaide, SA 5005 Australia

**Keywords:** Lower limb spasticity, Stroke, Botulinum toxin, Walking, Quality of life

## Abstract

**Background:**

Post-stroke lower limb spasticity (PSLLS) has a prevalence of 28–37%. PSLLS can cause difficulty in walking and reduce quality of life (QOL). Post stroke spasticity impairs the ability to intervene to improve walking ability. Botulinum Toxin A (BT) is an effective intervention for focal spasticity, but its use is currently restricted in many countries by their reimbursement system stating that the evidence for improvement in walking and quality of life (QOL) is not robust for treatment in the lower limb. This randomized control trial (RCT) will investigate the effectiveness of BT in modifying spasticity, and improving functioning (mobility, walking, activities of daily living (ADL’s) and QOL.

**Methods/design:**

A double-blind placebo-controlled trial injection will assess the effect of BT compared with a placebo (normal saline) in a sample of *n* = 94 patients. Following treatment of spasticity measured by Modified Ashworth Scale (MAS), the primary outcome of gait velocity will be measured by i) Gait Rite (Electronic Walkway); ii) walking by 2 Min Walk Test; iii) balance by Berg Balance Scale; mobility by iv) Timed Up and Go (TUG); v) lower limb function by ABILICO; vi) patient related goal by Goal Attainment Scale (GAS); vii) QOL by SF 12 (Rand version); viii) activities of daily living by the Functional Autonomy Measurement System (SMAF). There will be an associated health economic analysis.

**Discussion:**

The study methodology is based on our systematic review 2026 studies, which concluded the evidence for improving mobility following use of BT to reduce spasticity was not robust. The results of this study could establish the use of BT in improving gait and lower limb function in PSLLS. This study could provide the evidence needed for reimbursement schemes to consider and changes to its funding policy for BT in PSLLS.

**Trial registration:**

The trial is registered with the Australia New Zealand Clinical Trails Registry (ANZCTR)-ANZCTRN12617001603303. Registered 07/12/2017.

## Background

Stroke is a leading cause of death and the third most common cause of long-term adult disability in the developed world [1]. Stroke is Australia’s second biggest killer after coronary artery disease and a leading cause of disability. More than two thirds of stroke survivors develop impaired motor function and post-stroke spasticity. Post-stroke spasticity can significantly restrict stroke survivor’s mobility and functional ability, their activities of daily living and can diminish their quality of life. Motor impairments manifest as limitation of muscle control, body movements and associated functions, and typically affect one half of the body - the arm and the leg. The Stroke Foundation of Australia estimates there were 60,000 new and recurrent stroke cases in Australia in 2011 [2]. The cost of stroke burden is $2.14 billion a year. In recent decades, significant advances have taken place in diagnosis, management and prevention of stroke. However, stroke survivors continue to face great challenges due to long-term impairments and many lose their ability to live independently. Motor impairment is the most common and widely recognised impairment caused by stroke [3].

The prevalence of spasticity following stroke is between 17 and 46% [4]. Spasticity has been defined as “motor disorder characterized by velocity-dependent increase in tonic stretch reflexes (muscle tone) with exaggerated tendon jerks resulting from hyper excitability of the stretch reflex, as one component of the upper motor neuron syndrome.” [5, 6]. The effect of spasticity is profound. Following stroke, the body goes into safe mode (spasticity) causing loss of movement control, painful spasms, abnormal posture, increased muscle tone, and an overall decline in the function of muscles [7]. Langhammer [8], showed that post stroke motor impairments have a major impact on motor function, balance, gait, mobility, and activities of daily living, however, patients can improve with intensive exercise training [8]. The first problem to deal with, in achieving this, is the effects of spasticity (muscle stiffness) on muscle tone and flexibility. Spasticity interferes with or stops the exercise prescription. There is strong evidence that intensive exercise following a stroke can rewire the brain and has neuro-protective effects by activating specific neural circuits and increasing molecules that enhance synaptic plasticity [8]. Repetitive task specific exercise training will produce the behavioural experience, that is the most potent modulator of brain plasticity [9] and shown to improve outcomes such as balance, mobility, and in walking distance [8]. Post stroke exercise and motor re-education is therefore an important rehabilitation tool. There is, however good evidence that BT has clinical benefit in treating the mechanical effects of spasticity and in preparing the way for exercise training [10, 11]. The key to improving stroke outcomes is first; to deal with spasticity, then implement a structured exercise program and rehabilitation. This study will detail an intervention reducing lower limb spasticity followed by the structured exercise and rehabilitation program.

Our recent systematic review of 2026 studies of spasticity treatment, using BT in the lower limb, showed only five studies fitted our criteria of randomized controlled trials describing the efficacy of BT [12]. Even in these five studies there were major design problems including sample selection, study inclusion criteria, outcome measures, administration of BT, and methods of statistical analysis [13–17]. From the studies done to date on the lower limb, the evidence base for improvement of active functions such as gait velocity, walking distance and quality of life using BT for lower limb post stroke spasticity, is not robust.

The aims of this study is to investigate the primary outcome of gait velocity by Gait Rite (Electronic Walkway) and the secondary outcomes are measuring spasticity by Modified Ashworth Scale (MAS), walking by 2 Min Walk Test [18]; balance by Berg Balance Scale [19]; mobility by Timed Up and Go (TUG) [20]; lower limb function by ABILICO [21]; patient related goal by Goal Attainment Scale (GAS) [22]; QOL by SF 12 (Rand version); activities of daily living by the Functional Autonomy Measurement System (SMAF) [23].

## Methods and design

The study is a single center study based in Central Adelaide Local Health Network (CALHN) and operated out of 2 campuses: the Queen Elizabeth Hospital and the Hampstead Rehabilitation Centre. The design of the study is a double- blind, randomized, placebo-controlled trial (RCT) of one set of intramuscular injections of BT to the intervention group and one placebo to the control group.

### Participant recruitment and settings

Recruitment to the study will be through via referrals from.

1) Acute stroke units and stroke rehabilitation units of CALHN;

2) General practitioners; and.

3) Private specialists.

Recruitment will also be through promotional flyers distributed via the Stroke Foundation of South Australia.

### Ethics and trial registration

This trial has received the approval from the Hospital Research Ethics Committee (under Central Adelaide Local Health Network (Q20170509). The trial is registered with the Australia New Zealand Clinical Trails Registry (ANZCTR)-ANZCTRN12617001603303 [24].

### Inclusion criteria

- Male or female subjects aged 20 to 80 years of age are eligible for this study if they have suffered a stroke resulting in focal spasticity in the knee causing stiff knee and/or equinovarus deformity, and have a Modified Ashworth Scale (MAS) of spasticity of 3+ in the quadriceps, Gastrocnemius/Soleus, Tibialis Posterior, Flexor Digitorum Longus, Flexor Hallucis Longus, or with any difficulty in weight bearing on the leg or walking with reduced speed of gait following stroke will be included. Note will be made of the first stroke and/or previous strokes. All patients should have been walking (with a stick or without any aid) normally prior to stroke.

### Exclusion criteria

- Significant speech or cognitive impairment
- Significant lower limb problems such as fracture or arthritis, evidence of fixed contracture
- Use of BT type A in the previous six months, other non-stroke neurological disorders causing lower limb spasticity
- Significant illness, such as malignancy
- Contraindication to BT type A
- Pregnancy and lactation
- Osteoarthritic knee or hip having pain score of 3/10 or more on Visual Analog Scale
- Depression with the score of > 12/15 on Geriatric Depression Scale (65 years+) and score of > 30/63 on Beck Depression Inventory (under 65 years)
- Individuals on antispasticity medications such as Baclofen, Tizanidine, Dantrolene and Diazepam.

### Consent and randomization

Once eligible for the study, the participants will be provided with information about the study. After obtaining the informed consent, they will be randomized to either the intervention or the control group by the hospital pharmacist external to this project. We will be looking at the imaging (CT/MRI) to determine the type of stroke (Ischaemic vs Haemorrhagic) and there will be stratification within stroke types utilizing block permutation design. Rights of withdrawal without prejudice to future treatment at any time in the study will be emphasized.

### Outcomes

Primary outcome include change in

- Gait velocity/walking distance in GaitRite (Electronic walkway)

Secondary outcomes include changes in

- Spasticity measured by Modified Ashworth Scale (MAS)
- 2 Min Walk Test [18]
- Berg Balance Score [19]
- Timed Up and Go (TUG) [20]
- Lower limb function by ABILICO [21]
- Goal Attainment Scale (GAS) [22]
- Quality of life by SF 12 (Rand version)
- ADL score -The Functional Autonomy Measurement System (SMAF) [23]
- Passive range of motion at the ankle joint.
- Use of ActivPAL to quantify ambulatory activities at home [25]

### Procedures

**Fig. 1:**
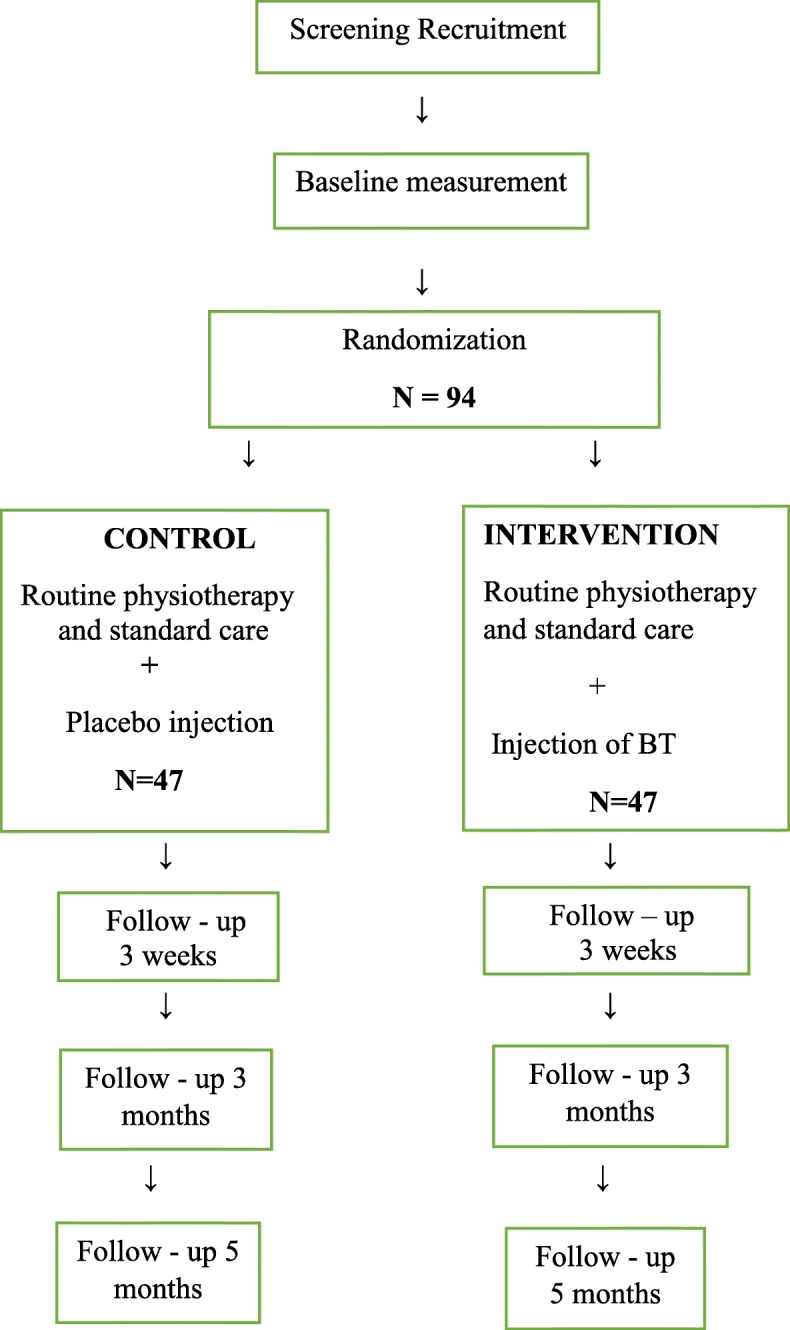
Study Design- Randomized Controlled Trial

Participants will undergo assessments at base line, 3 weeks, 3 months and 5 months following the injection (Fig. 1). All primary and secondary outcomes will be measured at all 4 time points. Additionally, the demographic and health characteristics such as age, gender, weight, type of stroke, affected side and mean time since stroke will he determined. A sensory evaluation will also be carried out in all the participants in the RCT.

We will also conduct a qualitative study described below. Both groups will receive standardized physiotherapy, including intensive exercise training, in terms of frequency, intensity and duration [8].

### BT and placebo administration to intervention and control

All treatment be it intervention or placebo (saline) will be constituted to the same type of syringes by the independent pharmacist. For all patients, physician will prescribe a dose of BT to be injected but will not be aware whether the syringe contains the active drug or placebo.

The intervention group will receive a targeted BT injection (up to 400 units of BT, Allergan, 1 vial will be reconstituted with 2 ml of normal saline) into the lower limb spastic muscles. The spastic muscles will be identified clinically and confirmed by EMG, (DANTEC CLAVIS). Maximum dose of Botox per session is 400 units (approved by TGA). The dose is dependent on the number of muscles and the size of the muscle involved. The most common deformity in post-stroke patients is equinovarus deformity. In an equinovarus deformity (caused by Gastrocnemius/soleus, Tibialis Posterior) the implicated muscles may require the following dosages. Gastrocnemius/Soleus-causing equinus deformity (plantarflexion)- may need up to 300 units. Tibialis Posterior – causing equinus and varus deformity- may need up to 200 units. Flexor Pollicis Longus and Flexor Digitorum Longus (causing clawing of great toe and other toes) - may need 100–150 units each. Patient specific dosage will be identified by the study physician at the start of the trial.

The pharmacist involved in randomization will constitute the dose into the same volume. The study physician will prescribe the dose but will get the same volume each time and won’t know if it is active or not. The Queen Elizabeth Hospital Pharmacy department will handle the medications and will prepare the identical looking blinded syringes.

### Routine physiotherapy and standard care

All participants in the study will receive physiotherapy and standard care such as orthotics (both in the intervention and the control group). This will include stretching, balancing, strengthening exercises and gait retraining. Gait retraining (task specific) will include walking on the level ground and then progressing to walking on inclines and different surfaces. Therapy will be standardized in terms of frequency (two sessions/week in the Physiotherapy department at the Queen Elizabeth Hospital, duration (45 min each session) and intensity (4–6 in Borg’s Scale of Perceived Exertion i.e. somewhat hard and hard). Patients will also be given a home exercise program. Compliance will be recorded monthly where participant activity will be recorded through phone calls. Additionally, phone calls will be made to encourage and motivate participants whereby they will be given advice about diet, weight and activity management. Activity at home will be monitored by ActivPal in sample subjects.

### Withdrawal criteria

Withdrawal criteria will cover subjects unable to comply with the study protocol, for any reason, or lost to follow up after 3 attempts of contact.

### Adverse events

Botulinum toxin has favorable safety and tolerability profile [26]. The list of adverse events will be looked for are i) injection site pain [27] ii) skin rash [28], and iii) transient muscle weakness or weakness observed beyond the site of local injection [29]. Potential for antibody formation can be minimized by using minimum effective dose and spacing the injection schedule [30]. In the event of any adverse events, the participant will be encouraged to contact the study physician. If noted, data will be collected regarding adverse effects. Rights of withdrawal without prejudice to future treatment at any time in the study will be emphasized.

### Qualitative evaluation

A qualitative study will be undertaken to understand the phenomena of participants experience of their condition/s and the impact of the intervention and how this differs between the participants in the control and intervention group. The qualitative study has an important function in understanding the participants established and changing experiences and outcomes. The findings from the qualitative data will enhance the findings from the quantitative data and may lead to new study questions [31, 32].

We plan to interview approximately 10 participants in each arm before intervention and follow up at 3 weeks and 5 months (*N* = 20) (or until data saturation is achieved) [33]. Every 4th participant will be invited in this semi structured interview. The first interview will start with broad questions about their condition/s and their effect; e.g. how they currently understand/perceive their condition, what limitations these impose on their lives and their expectation of the impact of the intervention. The second interview will focus on the immediate impact of the intervention. The third interview will focus on longer term impact. The person collecting and analyzing the data will be blinded to the study group.

### Sample size calculation

In most of the RCT’s included in our systematic review sample inadequacies were identified. Chief among these was the problem of inadequate sample calculations for repeated measures design. In repeated measures studies, within patient observations are correlated which often results in an estimate of the standard deviation that is an underestimate of the true standard deviation. Failure to account for this leads to an increased in the risk of a Type 1 error. In this study, we inflated our sample size by applying a design effect (DEFF) of 1.5 to account for the repeated measure design. Calculations were based on the requirement that effects be detected at the 5% alpha level with 90% statistical power. Based on available evidence, it was assumed that the clinically meaningful difference between the two treatment groups would be 0.13 points in gait speed with a standard deviation of 0.13. Based on these assumptions, a sample of 35 patients per group is required following the application of the design effect (*n* = 23 * DEFF). We have further inflated the sample size to *n* = 47 per group to account for an expected attrition rate of 25%.

### Statistical methods

An intention-to-treat (ITT) and per-protocol analysis is planned. The data will be summarized using means with standard deviations and medians with inter-quartile range. Counts and percentages will be used to describe categorical measures. Baseline characteristics will be assessed to identify any differences between treatment groups that would require adjustment in subsequent analyses. A linear mixed effects model will be applied to assess treatment effects over time. Pairwise, post hoc comparisons will be made using the Student t-test. All tests will be two-tailed and assessed at the 5% alpha level.

### Cost benefit analysis

To inform the economic evaluation of Botulinum toxin for post stroke spasticity, differences in quality of life will be assessed between the intervention and control group. There are potential spillover effects of improving patients’ quality of life on the quality of life of patients’ carers. Both patients and carers will be asked to complete the 12 question, SF12 survey at every clinic visit. These data can be converted to utility values to inform the estimation of quality adjusted life years (QALYs).

The cost data included in the economic evaluation will focus on costs incurred in public hospitals, as this patient group are unlikely to make significant use of private hospitals. Patients will be asked for consent to access identified cost and activity data through SA Health. Patients and their carers will also be asked to describe their use of primary health care services in between clinic visits, to which unit costs will be attached to add to the cost analysis.

On completion of the trial, aggregate cost and QALY estimates will be generated for each patient. Differences in mean costs and QALYs will be used to generate the expected incremental cost per QALY gained. Bootstrapping will be used to represent the uncertainty around the mean result.

### Qualitative analysis

Interviews will be transcribed, and data analysis will be conducted using thematic analysis [31].

## Discussion

This RCT protocol is carefully designed for evaluating the effectiveness of BT in improving the exercise program outcomes in the intervention group compared to the control group with increased effects on walking and other lower limb functioning and improved QOL and ADLs.

Improved walking is one of the highest priorities for people living with stroke [34]. Walking is a mode of bipedal locomotion in which both feet remain in contact with the ground, followed by a period of single stance on one limb while the other limb is swung forward. Post stroke patients with equinovarus deformity are unable to achieve proper contact with the ground resulting in poor stance on the affected leg and lose the heel toe rhythm of walking due to plantarflexion/inversion of the foot. Most of the phases of gait such as initial contact, loading response, mid stance, push off (stance phase), initial swing, and mid swing (swing phase) are affected. The awkward positioning of the foot and spasticity impairs balance, transfer, stride, gait and mobility besides causing spasm and pain. The awkward positioning can also lead to fall and fractures [35]. There is significant human and economic cost of spasticity [36]. Inability to walk is a predictor of losing independence and/or discharge to a nursing home [37]. Above all spasticity contributes to a range of adverse health outcomes including mortality [38]. In the upper limbs, there is no related dependence of operation. If one side of the body is affected, following stroke, the other side can be trained to compensate. In the lower limb, the dependence of both legs is essential for standing, maintaining balance, transferring from sitting to standing position and walking ability. This emphasizes the fact that rehabilitation of the lower extremities is very important. This study will achieve measurable important methodological and statistical outcomes, which can be replicated by other studies. The study has the methodological rigor of a placebo control trial. All participants will be provided with the standard care. At the end of the study the BT and placebo treated participants will be offered BT injection.

This study will provide useful descriptive information relating to spasticity and its effects following stroke qualitatively and quantitatively. The study will then also provide information on the ability of BT to control spasticity in order to retrain participants walking ability, outcomes on activities of daily living and quality of life. Given a positive study result in improving lower limb function it will provide an evidence-based treatment option that can argue for changes in PBS policy in funding the use of BT for treatment of PSLLS.

## Abbreviations

ADL: Activities of Daily Living
ANZCTR: Australia New Zealand Clinical Trails Registry
BT: Botulinum Toxin
GAS: Goal Attainment Scale
ITT: Intention to treat
MAS: Modified Ashworth Scale
PSLLS: Post-stroke lower limb spasticity
QALS: Quality Adjusted Life Years. CALHN- Central Adelaide Local Health Network
QOL: Quality of Life
RCT: Randomized Control Trial
SMAF: Functional Autonomy Measurement System
TUG: Timed Up and Go
